# Development and Function of the Voltage-Gated Sodium Current in Immature Mammalian Cochlear Inner Hair Cells

**DOI:** 10.1371/journal.pone.0045732

**Published:** 2012-09-19

**Authors:** Tobias Eckrich, Ksenya Varakina, Stuart L. Johnson, Christoph Franz, Wibke Singer, Stephanie Kuhn, Marlies Knipper, Matthew C. Holley, Walter Marcotti

**Affiliations:** 1 Department of Biomedical Science, University of Sheffield, Sheffield, United Kingdom; 2 Department of Otolaryngology, Tübingen Hearing Research Center, Molecular Physiology of Hearing, University of Tübingen, Tübingen, Germany; Dalhousie University, Canada

## Abstract

Inner hair cells (IHCs), the primary sensory receptors of the mammalian cochlea, fire spontaneous Ca^2+^ action potentials before the onset of hearing. Although this firing activity is mainly sustained by a depolarizing L-type (Ca_V_1.3) Ca^2+^ current (*I*_Ca_), IHCs also transiently express a large Na^+^ current (*I*_Na_). We aimed to investigate the specific contribution of *I*_Na_ to the action potentials, the nature of the channels carrying the current and whether the biophysical properties of *I*_Na_ differ between low- and high-frequency IHCs. We show that *I*_Na_ is highly temperature-dependent and activates at around −60 mV, close to the action potential threshold. Its size was larger in apical than in basal IHCs and between 5% and 20% should be available at around the resting membrane potential (−55 mV/−60 mV). However, *in vivo* the availability of *I*_Na_ could potentially increase to >60% during inhibitory postsynaptic potential activity, which transiently hyperpolarize IHCs down to as far as −70 mV. When IHCs were held at −60 mV and *I*_Na_ elicited using a simulated action potential as a voltage command, we found that *I*_Na_ contributed to the subthreshold depolarization and upstroke of an action potential. We also found that *I*_Na_ is likely to be carried by the TTX-sensitive channel subunits Na_V_1.1 and Na_V_1.6 in both apical and basal IHCs. The results provide insight into how the biophysical properties of *I*_Na_ in mammalian cochlear IHCs could contribute to the spontaneous physiological activity during cochlear maturation *in vivo*.

## Introduction

Inner hair cells (IHCs) of the mature mammalian cochlea relay sound information transduced by mechano-sensitive channels [1] to the central nervous system with high temporal precision [2] via the coordinated release of neurotransmitter onto type I spiral ganglion neurons [3]. However, before the onset of hearing (P12–P13 in most altricial rodents) IHCs do not respond to sound but instead generate spontaneous Ca^2+^-dependent action potentials [4], [5], which have been shown to be sufficient to induce the fusion of vesicles at the cell pre-synaptic site [4], [6]. These early spontaneous action potentials could be involved in regulating a variety of cellular responses and refinement of downstream neural circuits in developing systems [7], [8****]. Although action potentials in IHCs are mainly due to the interplay between a Ca^2+^current [4], [9****], carried by Ca_V_1.3 Ca^2+^ channels [10], and a delayed rectifier K^+^ current [9], their shape has been shown to be modulated by additional transiently-expressed conductances. The SK2 current (*I*_SK2_) and the Na^+^ current (*I*_Na_) have been shown to play a key role in modulating the firing frequency (*I*_SK2_ [11]; *I*_Na_ [4]) and in sustaining repetitive evoked AP activity (*I*_SK2_: [12], [13]). Therefore, a role for *I*_Na_ in shaping spontaneous action potentials *in vivo* could be crucial for the general maturation of the mammalian cochlea. Furthermore, a differential expression of *I*_Na_ in apical and basal IHCs could directly contribute to the functional differentiation of the IHCs themselves [**13**].

Voltage-gated Na^+^ channels are complexes of an α subunit and, in most cases, auxiliary β-subunits [14]–[16****]. While the α-subunit determines the main properties of the Na^+^ channels (ion permeability and kinetics) the β subunits are able to modify their kinetics and voltage dependence. A Na^+^ current has been reported in cochlear hair cells of lower vertebrates and mammals [4], [17]–[19****] and vestibular hair cells [20]–[25****]. Although the kinetics and voltage-range at which *I*_Na_ is functionally available seem to vary between hair cell types, some variation is simply due to the different recording conditions used (e.g. room vs body temperature). Vestibular hair cells express a TTX-sensitive *I*_Na_ carried by Na_V_ 1.1 and Na_V_ 1.2 channel subtypes [21], [24****] and a TTX-insensitive current carried by Na_V_ 1.5 channels [24]. However, the nature of the Na^+^ channels in mammalian cochlear hair cells remains unknown. Moreover, we know very little about the possible contribution of *I*_Na_ at around the IHC resting membrane potential and whether its biophysical properties differ as a function of cell position along the cochlea. The aim of this study was to investigate the biophysical properties and nature of *I*_Na_ in apical and basal IHCs of the rat cochlea during immature development. All *I*_Na_ recordings, apart from some designed to investigate its temperature-dependence, were performed in near physiological conditions (body temperature and using a perilymph-like solution) to ensure a more realistic estimate of the biophysical properties.

## Results

**Figure 1:**
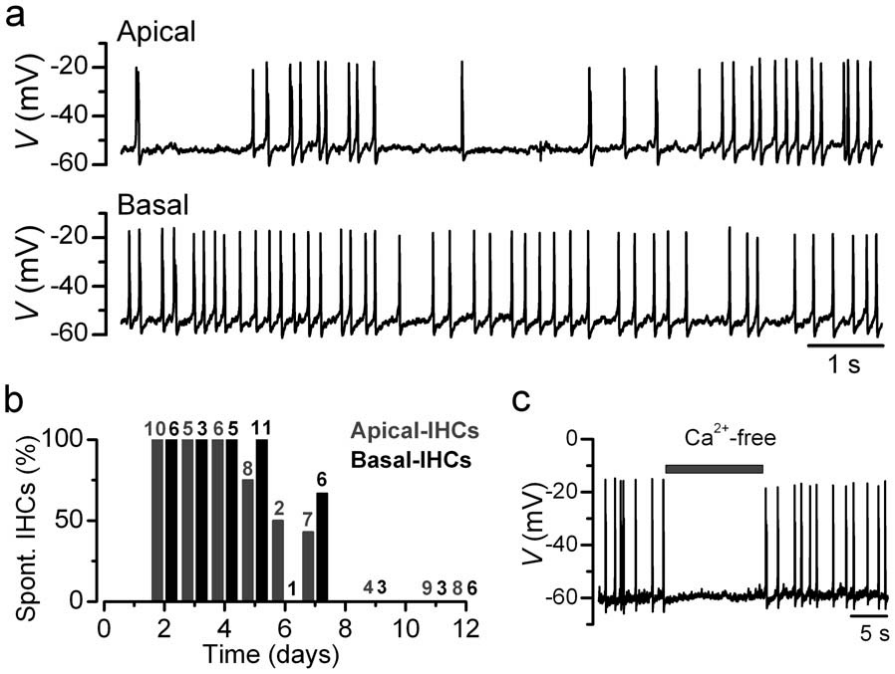
Spontaneous action potential activity of immature rat IHCs. **a**, Voltage responses from an apical-coil and a basal-coil P4 IHC with 1.3 mM Ca^2+^ in the extracellular solution. Note the more irregular spontaneous action potential activity in apical IHCs, in agreement with previous observations [5]. Cell properties were, apical: *V*_m_ −55 mV, *C*_m_ 8.9 pF, *R*_s_ 3.3 MΩ, *g*_leak_ 4.7 nS; basal: *V*_m_ −54 mV, *C*_m_ 8.3 pF, *R*_s_ 1.9 MΩ, *g*_leak_ 4.3 nS. **b**, Percentage of apical and basal IHCs found to be spontaneously active at the different postnatal age tested (P2–P12). Note that under the above recording conditions, no IHCs were found spontaneously active during the second postnatal week. Number of IHCs tested is shown above each bar of the histogram. **c**, Spontaneous action potentials are reversibly abolished during the superfusion of a nominally Ca^2+^-free solution. Apical P3 IHC: *V*_m_ −59 mV. Unless otherwise stated all recordings in this and following Figures are near body temperature (34–37°C).

Under near-physiological recording conditions (35–37°C and in perilymph-like extracellular solution containing 1.3 mM Ca^2+^ and 5.8 mM K^+^), apical and basal rat IHCs fired spontaneous action potential activity (**Fig. 1a**), which *in vitro* seemed to be restricted to the first postnatal week of development (**Fig. 1b**). Spontaneous action potentials in rat IHCs were dependent on Ca^2+^ since the application of a nominally Ca^2+^-free solution reversibly abolished them (**Fig. 1c**; see also [4], [5****]). Despite the Ca^2+^-dependence of spontaneous action potentials, immature IHCs additionally express a Na^+^ current (*I*_Na_) that modulates the frequency of the spontaneous firing rate (mice [4]).

**Figure 2:**
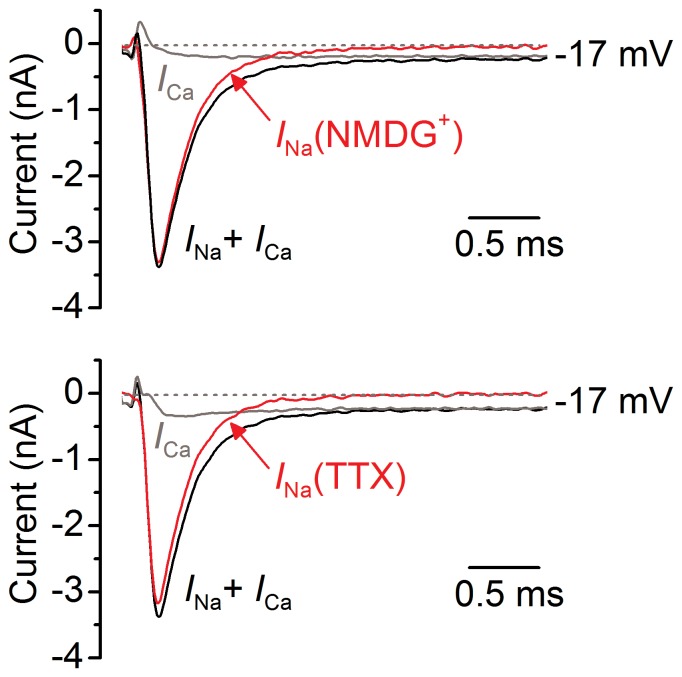
Inward sodium current in IHCs. Inward currents (*I*_Na_+*I*_Ca_: black traces) recorded from a P2 apical IHC obtained in the presence of K^+^ channel blockers. Recordings were obtained by applying a depolarizing voltage step to −17 mV from −110 mV. In this and the following figures actual test potentials, corrected for voltage drop across uncompensated *R*_s_, are shown next to the traces. Red traces show the isolated *I*_Na_ obtained by subtracting the current in the presence of NMDG^+^ (**a**: grey trace) or TTX (**b**: grey trace) from total inward current (black traces). The small residual current in NMDG^+^ or TTX (1 µM: grey traces) is the isolated *I*_Ca_. *C*_m_ 7.7 pF, *R*_s_ 0.7 MΩ, *g*_leak_ 1.6 nS.

The inward current carried by Ca^2+^ and Na^+^ (**Fig. 2**, black lines) was isolated from the total membrane current by reducing the much slower outward K^+^ currents present in immature IHCs with a Cs^+^-based intracellular solution. Since the Ca^2+^ current in IHCs is difficult to eliminate completely [10], we investigated *I*_Na_ in isolation by subtracting the current in the presence of NMDG^+^ or TTX from the total inward current (**Fig. 2**).

### Temperature dependence of the Na^+^ current in immature IHCs

**Figure 3:**
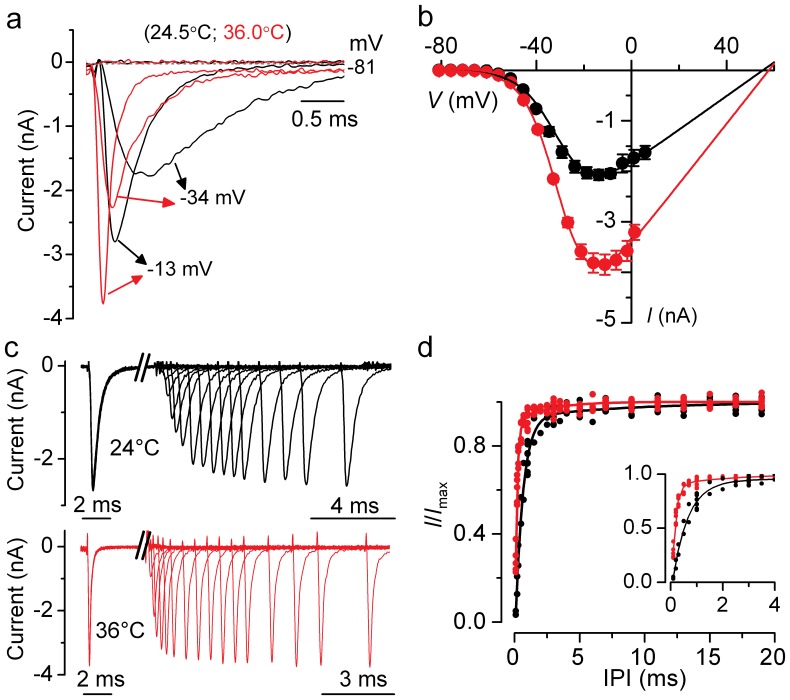
Na^+^ currents at body and room temperature. **a**, Isolated *I*_Na_ recorded from apical IHCs at body (red traces) and room (black traces) temperature. Currents were elicited by depolarizing voltage steps of 5 mV increments (10 ms in duration) following a 80 ms conditioning step to −110 mV in order to remove *I*_Na_ inactivation (holding potential was −81 mV). For clarity the first 3 ms of some of the traces are shown. IHC at 36.0°C: *C*_m_ 10.2 pF, *R*_s_ 0.84 MΩ, *g*_leak_ 2.2 nS. IHC at 24.5°C: *C*_m_ 8.6 pF, *R*_s_ 1.14 MΩ, *g*_leak_ 1.28 nS. **b**, Average peak current-voltage (*I*–*V*) curves for the isolated *I*_Na_ obtained from 5 IHCs at room temperature (black) and 7 IHCs at body temperature (red), including those shown in **a**. The continuous lines are fits using eqn. 1 and the fitting parameters are: body temperature *g*_max_ = 60 nS, *V*_rev_ = 58 mV, *V*_½_ = −30 mV, *S* = 7.0 mV; room temperature *g*_max_ = 33 nS, *V*_rev_ = 55 mV, *V*_½_ = −30 mV, *S* = 7.6 mV. **c** and **d**, Recovery of *I*_Na_ inactivation recorded at body and room temperature. Na^+^ currents (**c**) were elicited in response to 10 ms depolarizing voltage steps to −21 mV from −131 mV with varying the interpulse interval between steps (IPI in ms: 0.1, 0.2, 0.3, 0.5, 0.7, 1, 1.5, 2, 2.5, 3, 3.5, 4, 5, 6, 7, 9, 11, 13, 15, 17, 19). For clarity not all traces are shown. IHCs as in **a**. The two time scales below the traces refer to the time before and after the axis brake. **d**, Double exponential fit to individual *I*/*I*_max_ values showing the recovery of *I*_Na_ from inactivation from 9 cells recorded at room temperature (black) and 8 cells at body temperature (red). Expanded time scale for the first 4 ms is shown in the inset.

Since *I*_Na_ has commonly been recorded in mammalian hair cells at room temperature (∼25°C) instead of body temperature (∼36°C), we measured the temperature dependence of its size and kinetics. A typical example of *I*_Na_ recorded in rat IHCs at the two temperatures is shown in **Fig. 3*A***. Recordings were made using voltage steps of 5 mV increments from −110 mV. The current-voltage (*I*-*V*) curves for *I*_Na_ (**Fig. 3b**) were fitted using the following equation:
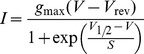
(1)where *I* is the current, *g*_max_ is the maximum chord conductance, *V* is the membrane potential, *V*_rev_ is the reversal potential of the current, *V*_½_ is the potential at which the conductance is half maximal activated and *S* is the slope factor that defines the voltage sensitivity of current activation. The maximum size of *I*_Na_ in apical IHCs, measured near the peak at −15 mV, was significantly smaller at 25°C (−2.1±0.1 nA, *n* = 7, P4) than at 36°C (−3.8±0.2 nA, *n* = 5, P3–P4: *P*<0.0001), giving a temperature sensitivity (Q_10_: see Materials and Methods) of 1.71 (ΔT used: 11°C). In both recording conditions, the *I*_Na_ activated at membrane potentials positive to −60 mV (defined as 1% of *g*_max_).

The time course of *I*_Na_ activation was measured as time to half maximal current amplitude and was found to be significantly faster (*P*<0.0005) at 36°C (0.180±0.006 ms, *n* = 16; measured for the maximal *I*_Na_ at −15 mV) than that at 25°C (0.227±0.004 ms, *n* = 5). The inactivation time constant (τ_inact_) of *I*_Na_ was evaluated by fitting the decay time course following the maximal current using a single exponential function. The τ_inact_ was significantly (*P*<0.0005) faster at 36°C (0.15±0.02 ms, *n* = 4; measured at −15 mV) than at 25°C (0.34±0.02 ms, *n* = 7). In order to investigate a possible difference in the rate of *I*_Na_ recovery from inactivation at 25°C and 36°C, apical IHCs were subjected to a two-pulse protocol in which they were depolarized to near −15 mV for 10 ms while changing the interpulse interval (IPI) from 0.1 ms to 50 ms (**Fig. 3c**). The relation between the normalized *I*_Na_ and IPI for both temperatures (**Fig. 3d**) was adequately described using a two exponential function. *I*_Na_ recorded at 36°C recovered from inactivation significantly faster (τ_fast_ = 0.14±0.01 ms; τ_slow_ = 2.30±0.47 ms, *n* = 8, P4) than that at 25°C (τ_fast_ = 0.58±0.03 ms; τ_slow_ = 8.77±2.77 ms, *n* = 9, P4; *P*<0.0005 and *P*<0.01, respectively). At both temperatures τ_fast_ was at least one order of magnitude more rapid than τ_slow_. It has been suggested that τ_slow_ represents *I*_Na_ recovery from a more inactivated state [**24**]. The above differences indicate that it is crucial to carry out physiological experiments at body temperature in order to understand the functional role of *I*_Na_ in mammals *in vivo*.

### Development of the Na^+^ current in IHCs

**Figure 4:**
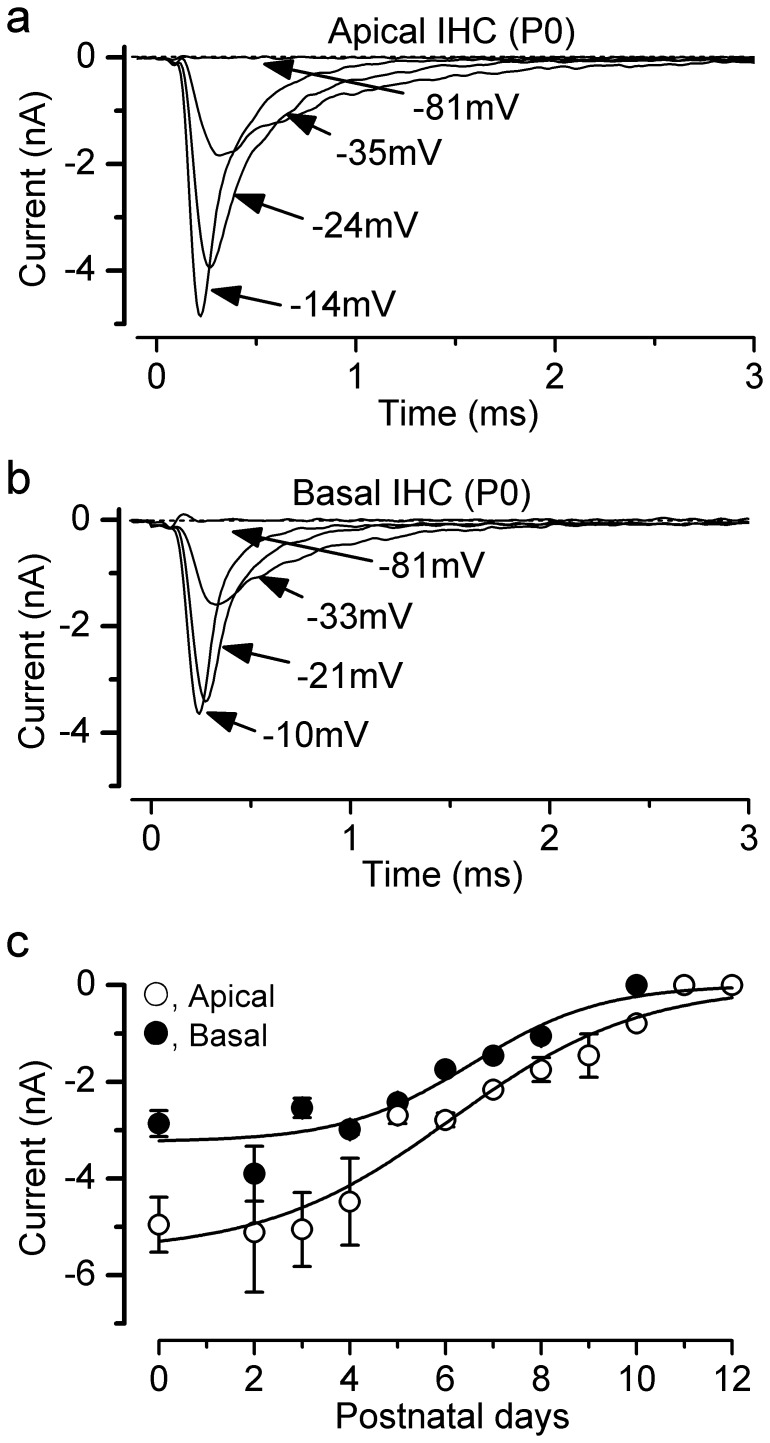
Changes in the size of the Na^+^ currents during IHC maturation at 36°C. **a**,**b**, Na^+^ current recorded from apical (**a**) and basal (**b**) P0 IHCs with the same voltage protocol described in **Fig. 3a**. For clarity only some of the traces are shown. Apical IHC: *C*_m_ 4.5 pF, *R*_s_ 1.2 MΩ, *g*_leak_ 0.6 nS. Basal IHC: *C*_m_ 7.1 pF, *R*_s_ 1.1 MΩ, *g*_leak_ 2. 6 nS. **c**, Development of the peak *I*_Na_ in apical- and basal-coil IHCs. Fits to the data are according to eqn. 2. Values for *t*_½_ and *k* are: apical: P6.1, 0.5 d^−1^; apical: P6.6, 0.7 d^−1^. Numbers of cells at the various ages (P0–P12) are: Apical 3, 0, 5, 6, 3, 3, 6, 1, 4, 3, 1, 5, 4; Basal 4, 0, 5, 5, 3, 2, 1, 1, 4, 0, 3, 3, 2.

We investigated the development of *I*_Na_ in IHCs from the apical and basal coils of the immature rat cochlea. Typical examples of *I*_Na_ are shown in **Fig. 4a,b**, respectively. Recordings were made using voltage steps of 5 mV increments from the holding potential of −110 mV. The developmental expression of *I*_Na_ was investigated between P0 and P12 by measuring its peak size near −15 mV (**Fig. 4c**). A large *I*_Na_ was already present at birth and from about P2 it began to decrease in size. By P10 in basal and P11 in apical IHCs *I*_Na_ was completely down-regulated. The data in **Fig. 4c** can be fitted to a sigmoidal logistic growth curve:
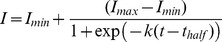
(2)


Where *I* is the size of the current, *k* is a slope factor and *t*_half_ is the age where *I* is halfway between the maximal (*I_max_*) and minimal (*I_min_*) currents. The size of *I*_Na_ was found to be significantly smaller (overall *P*<0.001, Two-way ANOVA: **Fig. 4c**) in basal than in apical IHCs.

### Properties of Na^+^ current activation and inactivation

**Figure 5:**
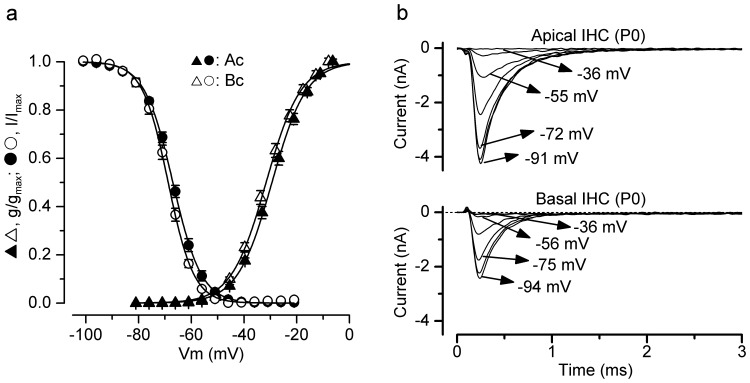
Activation and inactivation of the Na+ current in immature IHCs. **a**, Normalized activation and inactivation curves from P0–P4 apical IHCs. Continuous lines are calculated from eqn. 3 (activation) and eqn. 4 (inactivation). Fitting parameters for the activation curves are: apical IHCs (*n* = 14) *V*_½_ = −29.4 mV, *S* = 6.5 mV; *I*_max_ = −4289 pA; basal IHCs (*n* = 13) *V*_½_ = −31.6 mV, *S* = 6.6 mV; *I*_max_ = −3198 pA. Fitting parameters for the inactivation curves are: apical IHCs (*n* = 12) *V*_½_ = −67.0 mV, *S* = 5.4 mV; *I*_max_ = −4341 pA; basal IHCs (*n* = 10) *V*_½_ = −68.8 mV, *S* = 4.9 mV; *I*_max_ = −2687 pA. **b**, *I*_Na_ was recorded from apical and basal P0 IHCs during 10 ms depolarizing test steps to −21 mV, following 50 ms conditioning steps (not shown) from −131 mV to −1 mV in 5 mV increments and used to derive the inactivation curves. Some of the conditioning steps are shown next to the traces. Apical and basal IHCs are as in **Fig. 4a**,**b**, respectively.

The kinetic properties of *I*_Na_ and the voltage range at which it is available were investigated in both apical and basal IHCs at 36°C. The time course of *I*_Na_ activation was measured as time to half maximal current amplitude and found to be not significantly different between apical (0.180±0.006 ms, *n* = 16; measured near the peak of *I*_Na_ at −15 mV) and basal (0.180±0.005 ms, *n* = 14) IHCs. Steady-state activation and inactivation of *I*_Na_ is shown in **Fig. 5**. The activation curves (**Fig. 5a**: triangles) were obtained by calculating the normalized chord conductance from the *I–V* curves obtained for each apical and basal IHC. Activation curves were approximated by first-order Boltzmann fits:
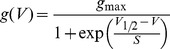
(3)where g is the chord conductance at membrane potential *V* and the other parameters are as in eqn. 1. *I*_Na_ activates for membrane potentials positive to about −60 mV (1% of *g*_max_). The half maximal activation (Apical: −29.4±0.18 mV, *n* = 14; Basal: −31.6±0.19 mV, *n* = 12) but not the slope factor (Apical: 6.54±0.16; Basal: 6.59±0.16 mV) was found to be significantly different (*P*<0.0001). Steady-state inactivation curves were obtained by measuring the peak *I*_Na_ (**Fig. 5b**) during a 10 ms test step to near −20 mV following a series of 50 ms conditioning steps to a range of voltages from −131 mV to −1 mV (5 mV nominal increments) from the holding potential of −81 mV. Normalized peak currents from 12 apical and 10 basal IHCs (P0–P4) were plotted against the different conditioning potentials (**Fig. 5a**: circles) and fitted by a first-order Boltzmann equation: 
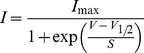
(4)where *I* is the peak current, *I*_max_ the maximal peak current and the other parameters are as in eqn. 1. Both the half inactivation (Apical: −67.0±0.09 mV; Basal: −68.8±0.12 mV) and the slope factor (Apical: 5.41±0.08 mV; Basal: 4.87±0.11 mV) were found to be significantly different between apical and basal IHCs (*P*<0.0001; *P*<0.005, respectively: fits to average data points). Depending on the age tested, IHCs have a resting membrane potential that varies between −50 mV and −60 mV, suggesting that between 5% and 20% of *I*_Na_ is available at rest.

### The sodium current modulates action potential activity

**Figure 6:**
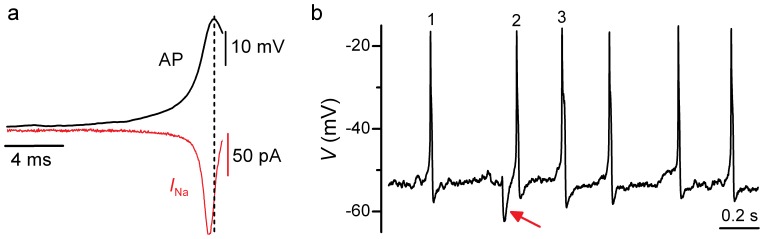
Role of the Na+ current in action potential activity. **a**, A spontaneous action potential (AP: top trace) was used as a voltage command to elicit *I*_Na_ (bottom trace) from the holding potential of −60 mV in four P2–P3 apical IHCs. The AP was recorded from an immature IHC under current clamp conditions at body temperature. **b**, Spontaneous action potential activity under current clamp recorded from a P2 basal IHC. The arrow indicates a spontaneous IPSP due to currents flowing through ACh receptors.

The Na^+^ current is able to shape action potentials and to facilitate firing frequency by speeding up the time necessary for the membrane potential to reach threshold [4]. In order to measure the degree to which *I*_Na_ contributes to shaping spontaneous action potentials, a voltage command mimicking a real action potential recorded from a spontaneously active cell was applied to P2–P3 apical IHCs. The size of the isolated *I*_Na_ (**Fig. 6a**) measured from the holding potential of −60 mV was −200±33 pA (*n* = 4), which corresponds to about 5% of the maximal available current (**Fig. 4c**). The peak *I*_Na_ occurred about 0.28±0.04 ms (*n* = 4) before that of the action potential waveform and it was almost completely inactivated while the cell membrane potential was still largely depolarized. Interestingly, we found that spontaneous hyperpolarizations (10.6±0.8 mV, *n* = 15, ranging between 5 mV and 15 mV: arrows in **Fig. 6b**), which are inhibitory postsynaptic potentials (IPSPs) mediated by the cholinergic efferent fibres [11], sometimes occurred just before an action potential and were able to transiently hyperpolarize IHCs negative to −65 mV (in some IHCs down to around −70 mV). The action potentials that followed IPSPs seemed to depolarize faster than the others. The small number of IPSPs preceding an action potential and the variability in the spike width in IHCs made it quite challenging to verify the above hypothesis. However, some evidence was obtained by measuring the width of action potentials between 20% and 80% of the spike height, which is the main place of action of *I*_Na_ (**Fig. 6a**). We compared the 20% to 80% width of spikes occurring immediately after an IPSP (spike 2 in **Fig. 6b**) to that of normal spikes (average of spikes 1 and 3: in **Fig. 6b**). We found that the subthreshold and upstroke width was significantly (*P*<0.005) smaller in action potentials with IPSPs (22.6±2.2 ms, *n* = 5, P2–P4) than in normal spikes (48.0±5.0 ms, *n* = 5). In the presence of the Na^+^ channel blocker TTX (1 µM), the 20%–80% spike width was not significantly affected by the preceeding IPSPs, indicating that the faster action potential depolarization following an IPSP is due to the TTX sensitive Na^+^ channels.

### Sodium channel subunits present in rat IHCs

**Figure 7:**
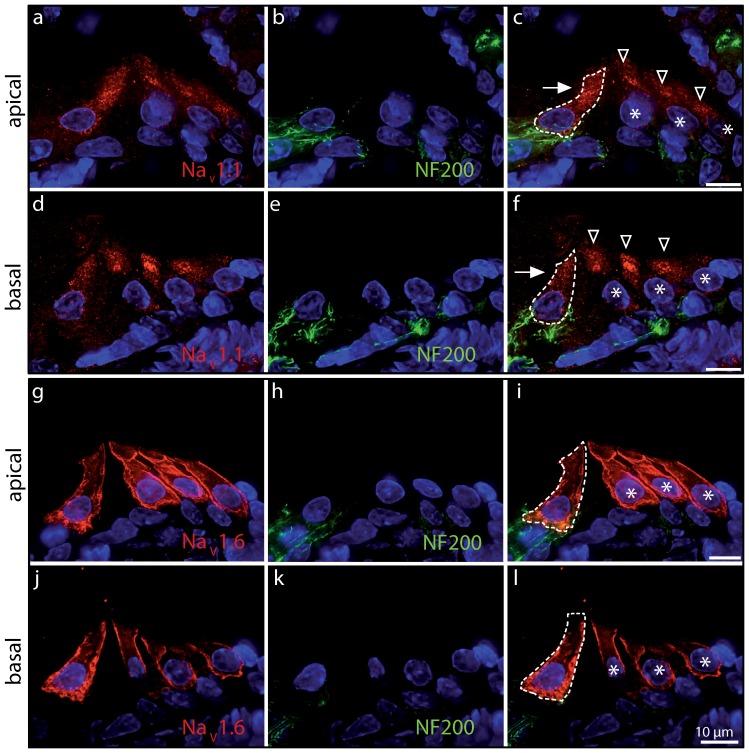
Na_V_ 1.1 and Na_V_ 1.6 are expressed in immature cochlear hair cells. Apical (**a**–**c**, **g**–**i**) and basal (**d**–**f**, **j**–**l**) cochlear turns of immature rat (P5) immunolabeled with antibodies against Na_V_ 1.1 (**a**, **d**; red) and Na_V_ 1.6 (**g**, **j**; red). Note that both IHCs and outer hair cells (OHCs) are labeled. Anti-Na_V_ 1.1 antibody labeling was mainly found in the supranuclear region of IHCs (arrow) and OHCs (arrowheads) and less intensely present in the cell membrane, which could reflect a high rate of channel turnover. Labelling was absent in adult IHCs (P20: data not shown), which do not have a Na^+^ current. Labeling for Na_V_ 1.6 was more obviously located to the membrane in both cell types. Co-labeling with anti-neurofilament antibody (NF200, green) showed no expression of Na_V_ 1.1 or Na_V_ 1.6 in afferent nerve fibres. Scale bars represent 10 µm. Cell nuclei are marked by DAPI (blue).

**Figure 8:**
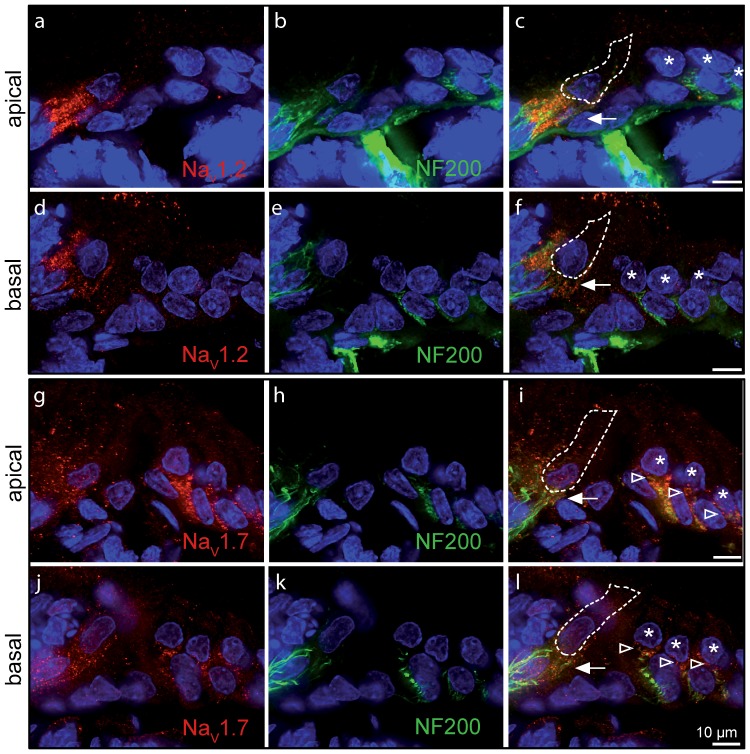
Na_V_1.2 and Na_V_1.7 are absent from immature IHCs. Apical (**a**–**c**, **g**–**i**) and basal (**d**–**f**, **j**–**l**) turns of immature (P5) rat cochlea. Antibodies to Na_V_ 1.2 and Na_V_ 1.7 (red) mainly labeled nerve fibres below IHCs (arrow) and OHCs (arrowheads), and colocalized with anti-neurofilament antibody (NF200, green). Scale bars represent 10 µm. Cell nuclei are marked by DAPI (blue).

Finally, we investigated the presence of TTX-sensitive Na^+^ channels (Na_V_1.1; Na_V_1.2; Na_V_1.3; Na_V_1.6; Na_V_1.7) in immature IHCs using immunolabeling experiments. We found that antibodies directed against Na_V_ 1.1 and Na_V_ 1.6 labeled apical and basal IHCs specifically within the organ of Corti (**Fig. 7**). By contrast, antibodies directed against Na_V_ 1.2 and Na_V_ 1.7 labeled the nerve fibres contacting IHCs (**Fig. 8**). Staining with a Na_V_1.3 antibody did not produce any labeling in our hands.

## Discussion

The aim of this work was to investigate the biophysical properties of *I*_Na_ at body temperature in apical and basal inner hair cells (IHCs) from the rat cochlea before the onset of hearing. This should provide insight into the physiological role of *I*_Na_ in shaping the electrical activity that is thought to guide hair cell differentiation and innervation. We also aimed to identify the type of α subunit carrying *I*_Na_. Although much of this is known for vestibular hair cells in various vertebrates, including mammals [20]–[26****], very little is known for mammalian cochlear hair cells.

### Na^+^ current in developing rat IHCs

Before the onset of hearing, immature IHCs fire spontaneous Ca^2+^-dependent action potentials [4], [5****]. Although these action potentials depend upon the activation of a Ca^2+^ current, their frequency is modulated by a transiently expressed Na^+^ current [**4**]. While only 71% of mouse IHCs seemed to express *I*_Na_ [4], all immature rat IHCs investigated showed a TTX-sensitive Na^+^ current. The reason for this difference between species is not known. The absence of a sustained component in the NMDG^+^-sensitive current (**Fig. 2a**) indicates that a “persistent” *I*_Na_, similar to that present in mammalian neurons [27] and in vestibular rat hair cells [24], is not present in cochlear IHCs. In rat IHCs, changing the recording conditions from ∼25°C to ∼35–37°C significantly increased the size of *I*_Na_ (Q_10_ = ∼1.7) and speeded up the activation and inactivation kinetics. The larger *I*_Na_ measured at near physiological temperature is likely to be caused by the increased Na^+^ channel conductance, which has been shown to increase with temperature by a Q_10_ of about 1.5 [28]. A similar temperature dependence has also been observed for the Ca^2+^ current expressed in mouse IHCs [**29]**, [30****]. At body temperature, the size of *I*_Na_ was larger in apical than in basal IHCs. During development, the maximal size of *I*_Na_ occurred between P0 and P4 (maximum current density −600 pA/pF) and then gradually decreased so that by P10 in basal and P11 in apical IHCs it was no longer detected. This is somewhat different from the development of *I*_Na_ in mouse IHCs, where it increases gradually up to P6 (maximum current density −141 pA/pF) whereupon it is gradually down-regulated [4]. Since *I*_Na_ recordings in mice and rats were performed using similar experimental conditions (e.g. body temperature) it seems that the time course and degree of expression of *I*_Na_ differs between rodents. Despite these differences, the biophysical properties of *I*_Na_ were very similar between rat and mouse IHCs.

### Na^+^ channel subunit composition in rat IHCs

In rat vestibular hair cells two types of *I*_Na_ have been identified with different sensitivity to TTX. Molecular analysis suggests that the TTX-insensitive *I*_Na_ is likely to be carried by Na_V_ 1.5 channels, while the TTX-sensitive current is carried by different subunits, including Na_V_ 1.2 and Na_V_ 1.6 [21], [24]. In cochlear IHCs *I*_Na_ is highly sensitive to TTX (*K*_D_ 4 nM [4]). Of the five neuronal TTX-sensitive Na^+^ channel isoforms (Na_V_ 1.1, Na_V_ 1.2, Na_V_ 1.3, Na_V_ 1.6 and Na_V_ 1.7 [16], [31], Na_V_ 1.7 has been suggested as the main carrier of *I*_Na_ in mouse IHCs [4] because of the similarity in its biophysical and pharmacological properties [32], [33]. However, in this study we found that the Na_V_ 1.7 protein was not detected in cochlear hair cells despite being present, together with the Na_V_ 1.2 subunit, in the nerve terminals (**Fig. 8**). This is consistent with previous findings showing the expression of Na_V_ 1.2 subunit in the efferent terminals on both IHCs and OHCs [34]. By contrast, the subunits expressed in IHCs and OHCs appeared to be Na_V_ 1.1 and Na_V_ 1.6 (**Fig. 7**). Although the biophysical properties of *I*_Na_ seem to be very similar between rat IHCs and OHCs (IHCs: this study; OHCs [19], their sensitivity to TTX appears to differ: ∼1 µM TTX fully blocked *I*_Na_ in IHCs (present study) but >10 µM was required in OHCs [19]. This discrepancy could be due to differences in the experimental manipulation between the two studies or to the differential expression of the Na_V_ 1.1 and Na_V_ 1.6 channels. However, we favour the first explanation for the following reasons. Co-expression of multiple Na^+^ channel isoforms has been described not only in neurons from the brain [35], [36] but also in hair cells [21], [24]. However, both Na_V_ 1.1 and Na_V_ 1.6 channel subunits have been reported to have a *K*_D_ for TTX in the order of a few nanomolar [16], which is similar to that reported *I*_Na_ in IHCs [4].

In the CNS, Na_V_ 1.1 is localized in the neuronal cell body [37] while Na_V_ 1.6 is generally found in dendrites and synapses [38]. Although in the cochlea both these isoforms seem to be expressed in the sensory hair cells, Na_V_ 1.6 shows a specific localization at the cell membrane that is reminiscent of that observed for the Ca_V_1.3 Ca^2+^ channels in immature IHCs [39], which are crucial for generating spontaneous action potentials. Na_V_ 1.1 was less intensely expressed in the immature IHC membrane compared to Na_V_ 1.6 (**Fig. 7**). Although both Na_V_ 1.1 and Na_V_ 1.6 play an important role in excitability, Na_V_ 1.6 is expressed in a specific population of motor neurons in the zebrafish that are required for activity-dependent developmental processes such as axonal outgrowth and pathfinding [40]. A similar role for Na_V_ 1.6 as a developmental regulator could also be present in IHCs since the Na^+^ current is directly involved in modulating the frequency of spontaneous Ca^2+^-dependent action potential activity [4], known to be able to influence IHC maturation [13].

### Physiological role of the Na^+^ current in pre-hearing rat IHCs

Spontaneous action potentials in pre-hearing IHCs are dependent on Ca^2+^, but the Na^+^ current decreases the time to reach the action-potential threshold [4]. Since *I*_Na_ shows steady-state inactivation, its main contribution to the action potential should depend on the IHC resting membrane potential, which in spontaneously active IHCs (P0–P7) varies between −55 mV and −60 mV. At body temperature, *I*_Na_ activates positive to −60 mV and half-inactivation occurs at −67 mV in apical and −69 mV in basal IHCs. Therefore, about 10–22% of *I*_Na_ in apical (average *I*_Na_ at P2 was −5.1±1.2 nA, *n* = 5) and 4–12% in basal (*I*_Na_ at P2 was −3.9±0.6 nA, *n* = 5) IHCs should be non-inactivated and thus available to shape the cell voltage responses. The presence of a functional *I*_Na_ at around the IHC resting membrane potential was confirmed by our voltage clamp experiments (**Fig. 6a**).

An important issue is to consider why IHCs express a large *I*_Na_ when only a small proportion is available from the resting membrane potential? A possible explanation could relate to the transient membrane hyperpolarizations, i.e. the inhibitory post-synaptic potentials (IPSPs), mediated by the cholinergic efferent fibres [11]. These IPSPs can delay or even prevent spontaneous action potentials [**11**]. However, we found that some IPSPs, which occurred just before an action potential (**Fig. 6b**), were able to transiently hyperpolarize IHCs down to as far as −70 mV. Since *I*_Na_ in IHCs has a very rapid recovery from inactivation (**Fig. 3d**), inhibitory synaptic potentials could be sufficient to recruit more than 60% of the current, thus substantially increasing the contribution of *I*_Na_ to the depolarization phase of an action potential. Indeed, the observed width of action potentials associated with IPSPs was narrower compared to those occurring in the absence of IPSPs. Although it is not known how the inhibitory efferent pathway normally operates *in vivo*, we have shown that the release of acetylcholine is likely to be more pronounced in the apex than in the base of the cochlea [5]. The larger size and availability of *I*_Na_ in apical IHCs (**Figs. 4** **,** 5), together with the more pronounced acetylcholine activity [5], indicates that *I*_Na_ is likely to have a major role in modulating the bursting-like firing pattern found in these apical cells, for example by promoting the return of spiking activity following a period of ACh induced hyperpolarization. This different modulation of action potential activity along the cochlea could be important to instruct the tonotopic differentiation of the IHCs themselves [41] as well as the possible refinement of tonotopic maps in the auditory pathway [42].

In this study we have shown that the biophysical properties of *I*_Na_ change during development and that the size and steady-state inactivation of *I*_Na_ differ between apical and basal IHCs. These differences could fulfil specific developmental requirements such as modulating spontaneous action potentials in pre-hearing IHCs. Moreover, we also provide the first evidence that *I*_Na_ in IHCs at this stage is carried by Na_V_ 1.1 and Na_V_ 1.6.

## Materials and Methods

### Ethics Statement

In the UK, all animal studies were licensed by the Home Office under the Animals (Scientific Procedures) Act 1986 and were approved by the University of Sheffield Ethical Review Committee. In Germany, care and use of the animals and the experimental protocol were reviewed and approved by the animal welfare commissioner and the regional board for scientific animal experiments in Tübingen.

### Tissue Preparation

Apical and basal coil inner hair cells (IHCs) from the rat were studied in acutely dissected organs of Corti from postnatal day 0 (P0) to P12, where the day of birth was P0. Rats were killed by cervical dislocation in accordance with UK Home Office regulations. Cochleae were dissected in extracellular solution (in mM): 135 NaCl, 5.8 KCl, 1.3 CaCl_2_, 0.9 MgCl_2_, 0.7 NaH_2_PO_4_, 5.6 D-glucose, 10 Hepes-NaOH. Sodium pyruvate (2 mM), MEM amino acids solution (50X, without L-Glutamine) and MEM vitamins solution (100X) were added from concentrates (Fisher Scientific, UK). The pH was 7.5 (osmolality ∼308 mosmol kg^−1^). The dissected cochleae were transferred to a microscope chamber and immobilized under a nylon mesh attached to a stainless steel ring. Extracellular solution was continuously perfused at a flow rate of about 30 ml/h (chamber volume was 2 ml) using a Masterflex L/S pump (Cole-Parmer, UK). The organs of Corti were viewed with an upright microscope (Leica DM-LFS, UK; Olympus BX51WI, UK) with Nomarski optics. The approximate position of IHCs along the cochlea was measured as the fractional distance from the extreme base. Recordings were made from IHCs located in the apical and basal coil of the rat cochlea, which correspond in adult animals to mean frequencies of about 3 kHz and 30 kHz, respectively, [43].

### Electrophysiology

Whole cell patch clamp recordings were mainly performed near body temperature (34–37°C). Some experiments were conducted at room temperature (25°C) in order to determine the effect of temperature on the biophysical properties of *I*_Na_. The temperature dependence of the size of *I*_Na_ was described by its temperature coefficient (Q_10_), which was calculated from the van’t Hoff equation: 
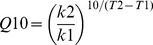
, where k1 and k2 are the values measured at the lower (T1) and higher (T2) temperatures, respectively.

Patch pipettes were made from either quartz (Sutter Instruments Co., USA) or soda glass (Harvard Apparatus Ltd, UK) capillaries and had a typical resistance in the extracellular solution of 2–5 MΩ. In order to reduce the electrode capacitance, the shank of the capillaries was coated with surf wax (Mr Zoggs SexWax, USA). The patch pipette filling solution used for current-clamp recordings contained (in mM): 131 KCl, 3 MgCl_2_, 1 EGTA-KOH, 5 Na_2_ATP, 5 Hepes-KOH, 10 sodium phosphocreatine (pH 7.3, 292 mosmol kg^−1^). For voltage-clamp recordings the solution was (in mM): 110 Cs-glutamate, 20 CsCl, 3 MgCl_2_, 1 EGTA-CsOH, 5 Na_2_ATP, 5 Hepes-CsOH, 10 Na_2_-phosphocreatine (pH 7.3, 294 mosmol kg^−1^). Current clamp experiments were made with an Optopatch amplifier (Cairn Research, UK) and voltage clamp recordings were made with an Axopatch 200B (Molecular Devises, USA). Data acquisition was performed using pClamp software with a Digidata 1320A or 1440A data acquisition board (Molecular Devises, USA). Voltage recordings were sampled at 5 kHz and low pass filtered at 2 kHz using (8-pole Bessel). Currents were sampled at 100 kHz and filtered at 10 kHz. Data were stored on a personal computer for offline analysis (Origin: OriginLab, USA; PClamp: Molecular Devices, USA). Membrane potentials in voltage clamp were corrected for the voltage drop across the uncompensated residual series resistance (*R*_s_: 1.2±0.1 MΩ, *n* = 72, after 70–90% compensation) and for a liquid junction potential, measured between electrode and bath solutions, of −11 mV for the Cs^+^-glutamate and −4 mV for the KCl intracellular solution. The average cell membrane capacitance was 7.6±0.1 pF (*n* = 72) and the average voltage-clamp time constant was 11±0.3 µs (*n* = 72).

IHC voltage recordings were performed in the presence of normal extracellular solution apart from a few recordings in which cells were locally superfused with a nominally Ca^2+^ free solution or concentrations of TTX (Tocris, UK) sufficient to fully block the Na^+^ current (≤1 µM [4]). In the Ca^2+^ free solution MgCl_2_ was increased to 3.9 mM to keep membrane charge screening approximately constant. The Na^+^ current was studied in isolation either by superfusing an extracellular solution containing TTX or by replacing Na^+^ with N-methyl-D-glucamine (NMDG^+^: Fluka, UK). The different superfused extracellular solutions were applied through a multi-barrel pipette positioned close to the patched hair cell.

Statistical comparisons were made by the two-tailed Student's *t*-test or, for multiple comparisons, two way ANOVA followed by a Bonferroni post-hoc test. Mean values are quoted ± s.e.m. (standard error of the mean) where *P*<0.05 indicates statistical significance.

### Immunocytochemistry

Immature rat (P5 and P20) cochleae were isolated, fixed, cryosectioned, and labeled for immunofluorescence microscopy as previously described [44], [45]. Briefly, cochleae were dissected and fixed for 2 hrs with 2% paraformaldehyde (wt/vol) or by injection of Zamboni's fixative [46] followed by 15 min incubation on ice. Then, cochleae were incubated overnight in 25% sucrose in phosphate-buffered saline (PBS), embedded in OCT compound and cryosectioned at a thickness of 10 µm. Sections were embedded with Vectashield mounting medium containg DAPI (Vector Laboratories, USA). Antibodies directed against Na_V_ 1.1, Na_V_ 1.2, Na_V_ 1.3, Na_V_ 1.6 and Na_V_ 1.7 (dilution 1∶50: Alomone Labs), and monoclonal mouse anti-neurofilament NF200 (dilution 1∶8000: Sigma) were used. Primary antibodies were detected with Cy3-conjugated (Jackson ImmunoResearch Laboratories, USA) or AlexaFluor 488-conjugated secondary antibodies (Molecular Probes, USA). Sections were viewed with an Olympus BX61 microscope equipped with epifluorescence illumination. Images were acquired with a CCD camera and analyzed with cellSens software (OSISGmbH, Germany). Figures show de-convoluted composite images, which represent the maximum intensity projection over all layers of the z-stack. Animals processed in Germany were asphyxiated with carbon dioxide before decapitation in accordance with the ethical guidelines approved by the University of Tübingen and the Tierschutzgesetz (Germany).
